# DNTTIP1 is a Prognostic Biomarker Correlated With Immune Infiltrates in Hepatocellular Carcinoma: A Study Based on The Cancer Genome Atlas Data

**DOI:** 10.3389/fgene.2021.767834

**Published:** 2022-02-21

**Authors:** Changyu Zhu, Rongsheng Tong, Xiaolei Jiang, Hua Xiao, Jianmei Guan, Jianchen Shu

**Affiliations:** ^1^ Department of Pharmacy, Sichuan Academy of Medical Science & Sichuan Provincial People’s Hospital, School of Medicine, University of Electronic Science and Technology of China, Chengdu, China; ^2^ Personalized Drug Therapy Key Laboratory of Sichuan Province, School of Medicine, University of Electronic Science and Technology of China, Chengdu, China; ^3^ Department of Pharmacy, Sichuan Academy of Medical Sciences & Sichuan Provincial People’s Hospital, Chengdu, China; ^4^ Department of Pharmacy, Gansu Provincial Hospital of TCM, Lanzhou, China; ^5^ Central Sterile Supply Department, Sichuan Academy of Medical Sciences and Sichuan Provincial People’s Hospital, Chengdu, China; ^6^ Department of Orthopedics, People’s Hospital of Leshan, Leshan, China

**Keywords:** DNTTIP1, HCC, tumor infiltration, prognosis, immune infiltrating

## Abstract

Deoxynucleotidyltransferase terminal-interacting protein 1 (DNTTIP1) is involved in the deacetylation of p53 in regulating cell cycle and is associated with cancers at the molecular level. In this study, we evaluated the prognostic value in hepatocellular carcinoma (HCC) based on data from The Cancer Genome Atlas (TCGA) database. Kruskal–Wallis test, Wilcoxon signed-rank test, and logistic regression were used to evaluate the relationship between DNTTIP1 expression and clinicopathological features. Cox regression and the Kaplan–Meier method were adopted to evaluate prognosis-related factors. Gene set enrichment analysis (GSEA) was performed to identify the key pathways related to DNTTIP1. The correlations between DNTTIP1 and cancer immune infiltrates were investigated by single-sample Gene Set Enrichment Analysis (ssGSEA). DNTTIP1 was found to be upregulated with amplification in tumor tissues in multiple HCC cohorts. High DNTTIP1 expression was associated with poorer overall survival (OS) and disease-free survival (DFS). GSEA suggested that DNTTIP1 regulates the cell cycle mitotic, G1/S, and G2/M phases and Fc fragment of IgE receptor I (FCERI)–mediated NF-κB and MAPK pathway and Fc fragment of IgG receptor (FCGR) activation pathways. Notably, ssGSEA indicated that DNTTIP1 expression was positively correlated with infiltrating levels of Th2 cells, Tfh, NK CD56 bright cells, aDCs, T helper cells, Th1 cells, and macrophages. These findings suggest that DNTTIP1 is correlated with prognosis and immune infiltration in HCC, which lays a foundation for further study of the immune-regulatory role of DNTTIP1 in HCC.

## Introduction

Hepatocellular carcinoma (HCC) is a serious medical problem that ranks sixth among the most common malignancies and is a third leading cause of cancer-related death (Forner et al., 2018). It has been reported that 80% of HCC cases occur in eastern Asia and sub-Saharan Africa where exposure to chronic hepatitis B (CHB) and aflatoxin B1 (AFB1) is the main risk factor (Forner et al., 2018). Over the past 10 years, treatment of HCC has considerably evolved. Today, HCC patients diagnosed at any stage of the disease can benefit from effective treatment that substantially improves their survival. However, several areas still need to be urgently improved. Deep sequencing in HCC has shown that the aggregation of driver and passenger gene alternations in the somatic genome and epigenetic modifications result in HCC (Schulze et al., 2016), which explains its huge molecular heterogeneity. Several candidate biomarkers are being studied in HCC; however, significant challenges exist largely stemming from HCC molecular heterogeneity (Sia et al., 2017; Sia and Llovet, 2017). There has been little progress made on the development of clinically useful biomarkers for early detection of HCC over the last 2 decades (Sengupta and Parikh, 2017). Furthermore, the molecular mechanisms underlying tumorigenesis and progression of HCC remain poorly understood (Zhao et al., 2019). Consequently, there is a critical gap in the current treatment and understanding of HCC due to absence of specific markers for tumor type or disease stage. Hence, the investigation of effective prognostic biomarkers is a pivotal area among several considerations within the research of HCC.

Deoxynucleotidyltransferase terminal-interacting protein 1 (DNTTIP1), that was first reported in 2001, is highly homologous to the transcription factor p65 (Yamashita et al., 2001). DNTTIP1 can enhance DNA polymerase activity (Motea and Berdis, 2010) and form a complex with histone deacetylase (HDAC) (Bantscheff et al., 2011); hence, it has a close relationship with cancer progression. For example, a recent study reported that DNTTIP1 expression could be a specific biomarker present in acute myelocytic leukemia (AML) (Zhuang et al., 2020). Previous research proved that DNTTIP1–HDAC interaction could deacetylate p53 and promote tumor growth in oral squamous cell carcinomas (OSCCs) (Sawai et al., 2018). Former studies have also shown the overexpression of individual HDACs and p53 in HCC (subtypes) and their impact on HCC progression (Ler et al., 2015; Liao et al., 2017; Freese et al., 2019). Based on previous studies, DNTTIP1 might be a promising biomarker of HCC. However, the relationship between DNTTIP1 and HCC has not yet been revealed.

## Materials and Methods

### Data Acquisition and Preprocessing

The RNA-seq data of 371 HCC and 50 normal tissues and patient clinical information were downloaded from the Liver Hepatocellular Carcinoma (LIHC) Project of The Cancer Genome Atlas (TCGA) (https://portal.gdc.cancer.gov/) (Blum et al., 2018) until 12 July 2020. Then, RNA-seq data in FPKM format were transferred to TPM (transcripts per million reads) format, retained, and further analyzed.

### Differentially Expressed Gene Analysis

We used the unpaired Student’s t-test within the DESeq2 R package (3.6.3) (Love et al., 2014) to compare the expression data (HTseq-Counts) between high- and low-expression groups according to the median DNTTIP1 expression level. The thresholds for the DEGs were |log2-fold change (FC)| >2.0 and adjusted *p* < 0.05.

### Enrichment Analysis

Metascape (3.0) (http://metascape.org), a user-friendly, well-maintained, free, gene list online analysis tool for gene analysis and annotation (Zhou et al., 2019), was adopted to perform Gene Ontology (GO) analysis. ClusterProfiler package in R (3.6.3) (Yu et al., 2012) was used to perform Gene Set Enrichment Analysis (GSEA) and detect the correlation between DNTTIP1 and the pathway. As a computational method, GSEA determines whether a priori defined set of genes have statistical significance and concordant differences in two biological states. The samples were divided into high- and low-expression groups according to the median expression level of DNTTIP1. DEseq was used to compare the different expressions between different groups. Gene set permutations were performed with 1,000 times random combinations for each analysis. In the whole process, the expression level of DNTTIP1 was regarded as a phenotype. Additionally, the adjusted P and normalized enrichment score (NES) were utilized to sort the enriched pathways in each phenotype (Subramanian et al., 2005). c2.cp.v7.0.symbols.gmt [Curated] in MSigDB collections was selected as a reference gene set. Gene sets with a false discovery rate (FDR) < 0.25 and adjusted *p* < 0.05 were considered significantly enriched.

### Immune Infiltration Analysis by Single-Sample Gene Set Enrichment Analysis

SSGSEA classifies marker gene sets in a single sample with common biologic functions, chromosomal localization, and physiological regulation. In this study, the ssGSEA method was realized by the GSVA package (Hänzelmann et al., 2013) in R to analyze the immune infiltration for 24 types of immune cells and correlation between DNTTIP1 and every immunocyte in HCC samples according to the published literature (Bindea et al., 2013). The relative enrichment score of signature genes was quantified from the gene expression profile for each tumor sample. Spearman’s correlation was adopted to analyze the correlation between DNTTIP1 and 24 types of immune cells, and the Wilcoxon rank-sum test was adopted to analyze the infiltration of immune cells between the high-expression groups of DNTTIP1.

### Protein–Protein Interaction Network

The Search Tool for the Retrieval of Interacting Genes (STRING) database (http://string-db.org) (Szklarczyk et al., 2019) was applied to predict the PPI network. The combined score threshold of interaction was 0.4. Furthermore, we extracted the hub genes in this PPI network by using MCODE to identify crucial subnetworks and visualize the PPI network by using Cytoscape (version 3.7.2).

### Statistical Analysis

The statistical data acquired from TCGA were merged and processed by R 3.6.3. The Wilcoxon rank-sum test and Wilcoxon signed-rank test were used for comparing the expression levels of DNTTIP1 between HCC and the control group. Kruskal–Wallis test, Wilcoxon rank-sum test, Wilcoxon signed-rank test, and Spearman’s correlation were used to analyze the relation between DNTTIP1 expression and grade of clinicopathological factors. Normal and adjusted Pearson’s κ^2^ test, Fisher’s exact test, and univariate logistic regression were used to analyze whether the grade of clinicopathological factors affects DNTTIP1 expression. Spearman’s correlation and the Wilcoxon rank-sum test were adopted to analyze the infiltration of immunocytes between the high- and low-expression groups of DNTTIP1. Comparison of multiple groups was performed using a nonparametric Kruskal–Wallis test followed by a post hoc Dunn’s test with Bonferroni correction for pairwise comparisons. Univariate Cox regression analysis and multivariate Cox regression analysis were used to evaluate the influence of DNTTIP1 expression and other clinicopathological factors (age and gender) on survival. The significant variables in the univariate analysis (*p* < 0.1) were included into the multivariate analysis (Boeck et al., 2013; Fischer-Rasokat et al., 2021). The Kaplan–Meier curve was drawn to evaluate the prognostic value of DNTTIP1. Hazard risk (HR) of individual factors was estimated by measuring the HR with a 95% confidence interval (CI).

Receiver operating characteristic (ROC) analysis was performed by the pROC package (Robin et al., 2011). The calculated area under the curve (AUC) value ranges, which were from 0.5 to 1.0, indicated the discrimination ability of 50%–100%. We constructed a nomogram by the rms R package based on the results of the multivariate analysis. The predicted survival probability for 1, 3, and 5 years is visualized in the nomogram, which includes a calibration plot as well as significant clinical characteristics. All statistical tests were considered significant when two-tailed *p* ≤ 0.05.

## Results

### Clinical Characteristics

The clinical data of 371 HCC patients included patient age, gender, T stage, N stage, M stage, pathologic stage, histologic grade, fibrosis Ishak score, vascular invasion, tumor status, TP53 status (%), age, alpha-fetoprotein (AFP) (ng/ml), and prothrombin time (Table 1). A total of 250 males and 121 females with a mean age of 61 years were analyzed in the present study, including 184 white patients and 175 non-white patients. The chi squared test showed that DNTTIP1 was significantly correlated with fibrosis Ishak score (*p* = 0.023), vacuum invasion (*p* = 0.035), and TP53 status (*p* < 0.001). Fisher’s exact test showed that DNTTIP1 was significantly correlated with histologic grade (*p* < 0.001). Wilcoxon rank sum test showed that DNTTIP1 was significantly correlated with weight (*p* = 0.047), AFP (ng/ml) (*p* < 0.001), and prothrombin time (*p* = 0.022). DNTTIP1 expression was not significantly correlated with other clinicopathological features.

**TABLE 1:** Demographic and clinicopathological parameters of patients with hepatocellular carcinoma in TCGA–LIHC.

Characters	Level	Low expression of DNTTIP1	High expression of DNTTIP1	*p*
N		186	185	
T stage (%)	T1	100 (54.6%)	81 (43.8%)	0.146
	T2	43 (23.5%)	51 (27.6%)	
	T3	36 (19.7%)	44 (23.8%)	
	T4	4 (2.2%)	9 (4.9%)	
N stage (%)	N0	125 (99.2%)	127 (97.7%)	0.622
	N1	1 (0.8%)	3 (2.3%)	
M stage (%)	M0	126 (97.7%)	140 (99.3%)	0.351
	M1	3 (2.3%)	1 (0.7%)	
Pathologic stage (%)	Stage I	94 (53.7%)	77 (44.8%)	0.141
	Stage II	41 (23.4%)	45 (26.2%)	
	Stage III	36 (20.6%)	49 (28.5%)	
	Stage IV	4 (2.3%)	1 (0.6%)	
Histologic grade (%)	G1	35 (19.0%)	20 (11.0%)	<0.001
	G2	100 (54.3%)	77 (42.3%)	
	G3	47 (25.5%)	75 (41.2%)	
	G4	2 (1.1%)	10 (5.5%)	
Gender (%)	Female	65 (34.9%)	56 (30.3%)	0.395
	Male	121 (65.1%)	129 (69.7%)	
Fibrosis Ishak score (%)	0	52 (42.6%)	22 (24.4%)	0.023
	1/2	16 (13.1%)	15 (16.7%)	
	3/4	11 (9.0%)	17 (18.9%)	
	5/6	43 (35.2%)	36 (40.0%)	
Vascular invasion (%)	No	116 (71.2%)	90 (59.2%)	0.035
	Yes	47 (28.8%)	62 (40.8%)	
Tumor status (%)	Tumor-free	103 (59.2%)	98 (55.1%)	0.499
	With tumor	71 (40.8%)	80 (44.9%)	
TP53 status (%)	Mut	28 (15.3%)	74 (42.3%)	<0.001
	WT	155 (84.7%)	101 (57.7%)	
Age (median [IQR])		61.00 [53.00, 68.75]	61.00 [51.00, 69.00]	0.929
AFP(ng/ml) (median [IQR])		7.50 [3.00, 46.00]	35.50 [7.00,1779.50]	<0.001
Prothrombin time (median [IQR])		1.10 [1.00, 10.00]	1.10 [1.00, 1.30]	0.022

### Identification of Differentially Expressed Genes in Hepatocellular Carcinoma

**FIGURE 1:**
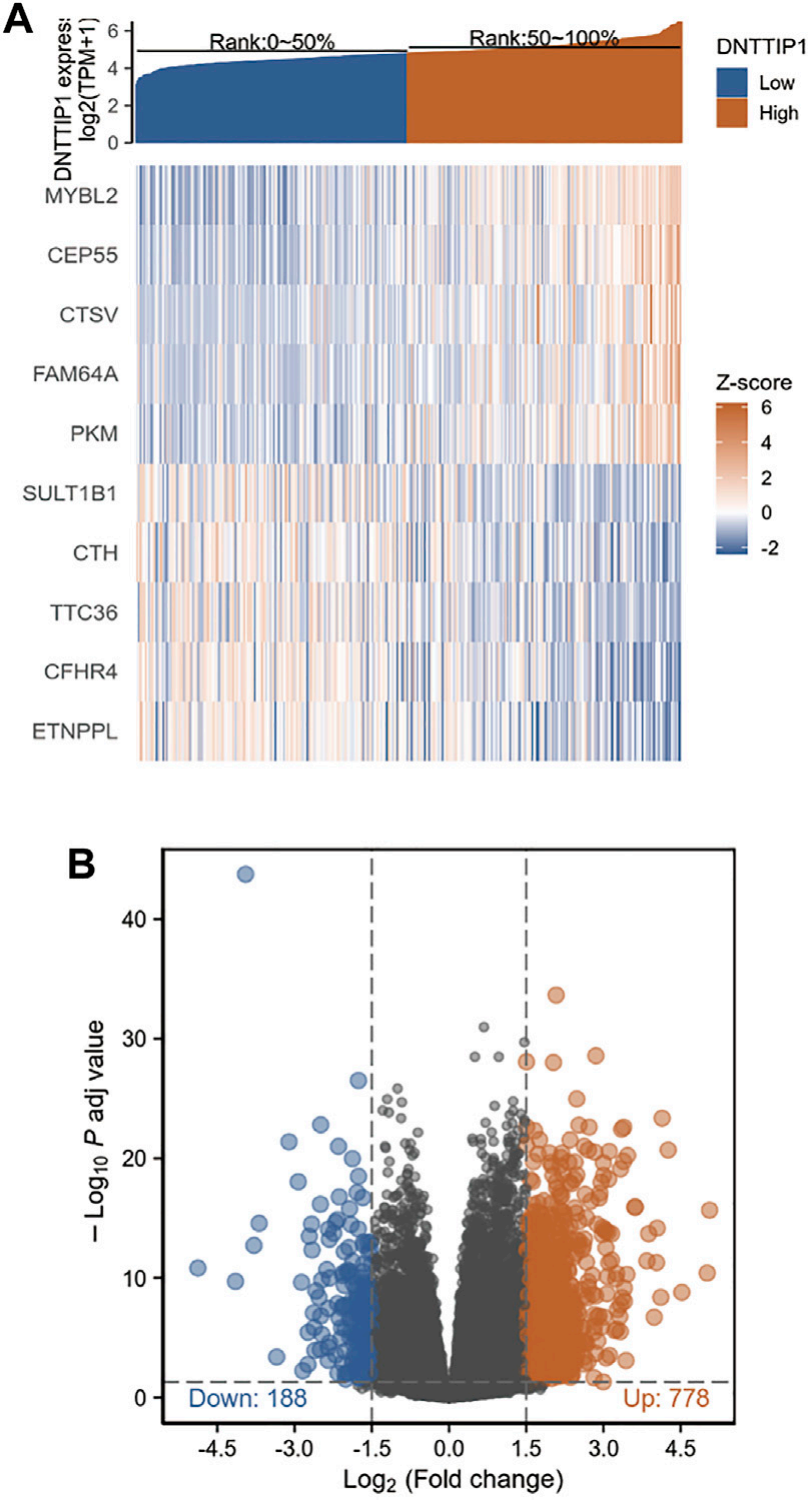
Results of differentially expressed gene (DEG) analysis. **(A)** Heat map of the 10 differentially expressed genes. **(B)** Volcano plot of differentially expressed RNAs.

Based on the cutoff criteria (|logFC| <1.5 and adjusted *p* < 0.05), we identified a total of 966 DEGs (778 upregulated and 188 downregulated) after using the DESeq2 package in R (Love et al., 2014) to analyze the HTSeq-count data from TCGA. DEG expressions were illustrated by a heat map and volcano plot (Figures 1A,B). DEGs included 812 differentially expressed RNAs (651 upregulated and 161 downregulated), which contained 560 mRNAs (462 upregulated and 98 downregulated) and 252 lncRNAs (189 upregulated and 63 downregulated) (Supplementary Figures S1A,B).

### Functional Enrichment Analysis of Differentially Expressed Genes

**FIGURE 2:**
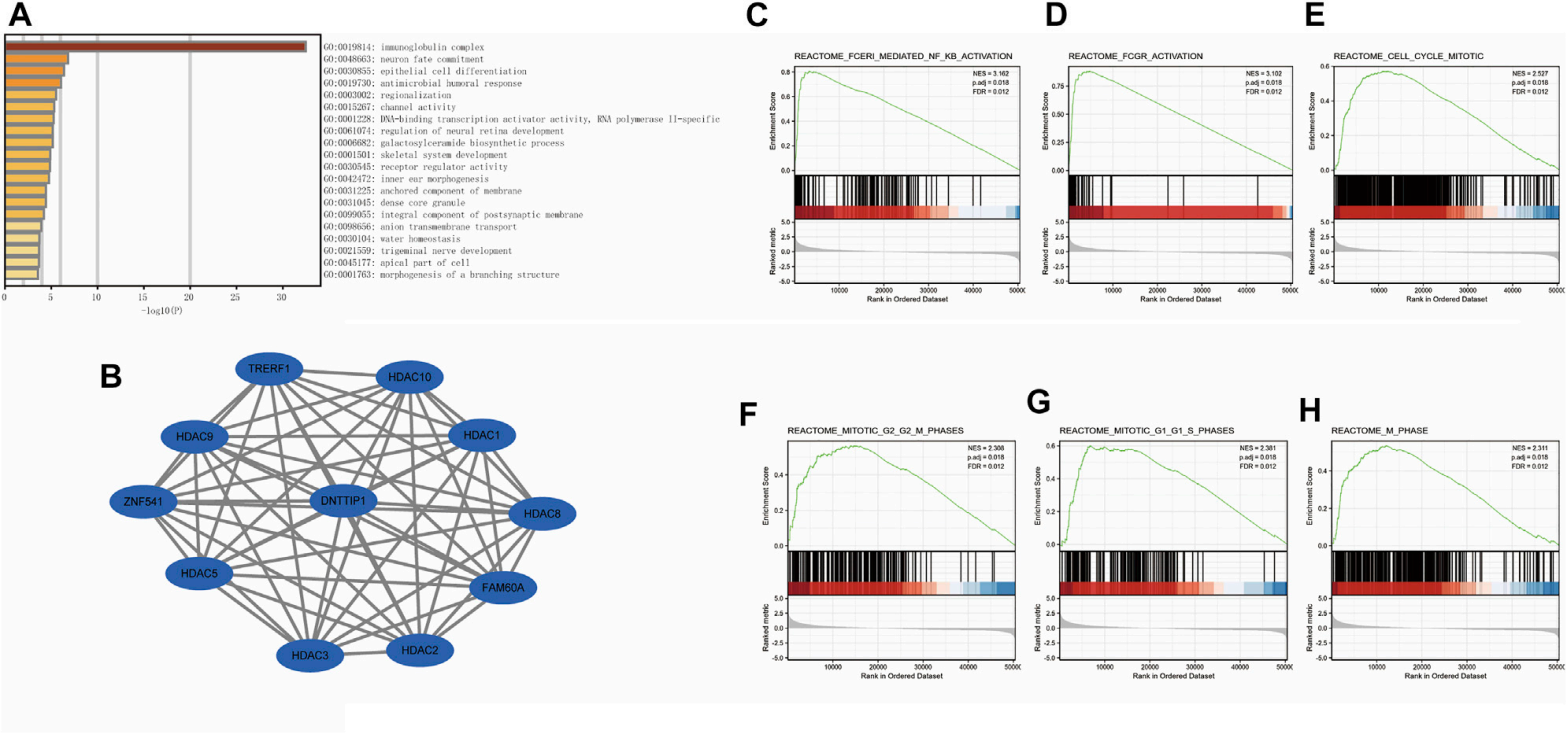
Enrichment analysis of DNTTIP1 in HCC. **(A)** Top 20 biological process enrichment related to DNTTIP1-related genes with enrichment heatmap. **(B)** Network of DNTTIP1 and its potential coexpression genes in DNTTIP1-related DEGs. **(C–H)** Results of enrichment analysis from GSEA.

We used Metascape to perform GO enrichment analyses of the functions of DNTTIP1-associated DEGs in HCC. The GO results displayed that DNTTIP1-associated DEGs had significant regulation on immunoglobulin complex, neuron fate commitment, epithelial cell differentiation, neuron fate commitment, antimicrobial humoral response, regionalization, channel activity, DNA-binding transcription activator activity, RNA polymerase II-specific, galactosylceramide biosynthetic process, and regulation of neural retina development. Moreover, galactosylceramide biosynthetic process, skeletal system development, receptor regulator activity, inner ear morphogenesis, anchored component of membrane, dense core granule, an integral component of postsynaptic membrane, anion transmembrane transport, water homeostasis, trigeminal nerve development, apical part of the cell, and morphogenesis of a branching structure were also involved in regulating DNTTIP1-interacting genes (Figure 2A). A network of DNTTIP1 and its potential coexpression genes in DNTTIP1-related DEGs are shown in Figure 2B. DNTTIP1-related signaling pathways identified by GSEA are shown in Figures 2C–H.

As many pathways contribute to tumor formation, high DNTTIP1 expression associated with poor survival may be related to active signaling pathways in HCC. We performed GSEA of differences between low- and high–DNTTIP1 expression data sets to identify the key signaling pathways associated with DNTTIP1. A total of 476 pathways showed significant differences (FDR<0.05, adjusted *p* < 0.05) in the enrichment of the MSigDB collection (c2.cp.v7.0.symbols). The most significantly enriched signaling pathways based on their NES are shown in Table S1. In particular, DNTTIP1 was related to the cell cycle mitotic, G1/S, and G2/M phases in the cell cycle and Fc fragment of IgE receptor I (FCERI)–mediated NF-κB and MAPK pathway and Fc fragment of IgG receptor (FCGR) activation (Figures 2C–H).

### The Correlation Between DNTTIP1 Expression and Immune Infiltration

**FIGURE 3:**
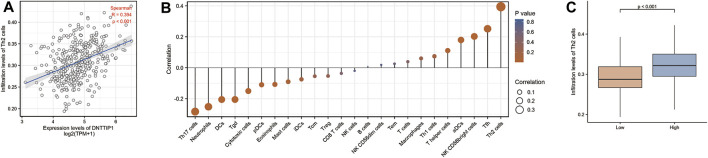
Results of analysis between DNTTIP1 expression and immune infiltration. **(A)** Th2 cells were significantly positively correlated with DNTTIP1 expression. **(B)** Correlation between the relative abundances of 24 immune cells and DNTTIP1 expression level. The color of dots shows the absolute value of Spearman R. **(C)** Th2 cell infiltration level in different DNTTIP1 expression groups.

We employed Spearman’s correlation to show the association between the expression level (TPM) of DNTTIP1 and immune cell infiltration level quantified by ssGSEA in the HCC tumor microenvironment. Th2 cells were significantly positively correlated with DNTTIP1 expression (Spearman R = 0.394, *p* < 0.001) (Figure 3A). Other immune cell subsets, including Tfh, NK CD56 bright cells, aDCs, T helper cells, Th1 cells, and macrophages, were also correlated with DNTTIP1 expression (Figure 3B). The Th2 cell infiltration level in the DNTTIP1 high-expression group was significantly different from that of the low-expression group (*p* < 0.001) (Figure 3C).

### Associations Between DNTTIP1 Expression and Clinico-Pathologic Variables

**FIGURE 4:**
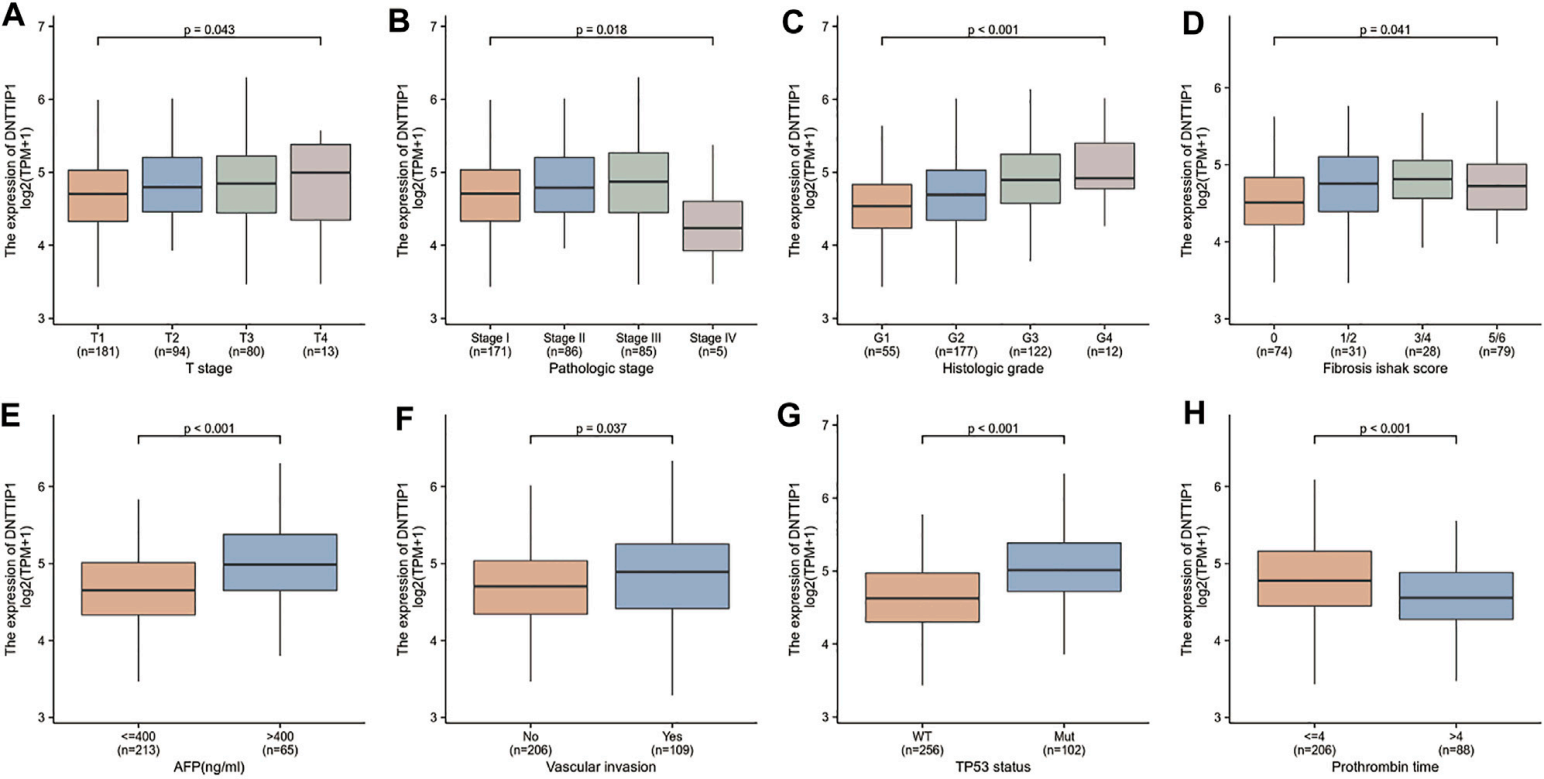
Association between the DNTTIP1 expression and different clinicopathological characteristics. **(A)** Association between the DNTTIP1 expression and T stage of HCC, **(B)** pathologic stage, **(C)** histologic grade, **(D)** fibrosis Ishak score, **(E)** AFP, **(F)** vascular invasion, **(G)** TP53 status, and **(H)** prothrombin time.

The Kruskal–Wallis rank-sum test revealed that the expression of DNTTIP1 was significantly correlated with the T stage of HCC (*p* = 0.049). Pathologic stage (*p* = 0.015), histologic grade (*p* < 0.001), fibrosis Ishak score (*p* = 0.008) (Figures 4A–D), and Wilcoxon rank-sum test revealed that expression of DNTTIP1 was significantly correlated with AFP(*p* < 0.001), vascular invasion (*p* = 0.037), TP53 status (*p* < 0.001), and prothrombin time (*p* < 0.001) (Figures 4E–H). Bonferroni correction was applied to the *p* value of Dunn’s test to correct for multiple comparisons within the T stage of HCC. Pathologic stage, histologic grade, and fibrosis Ishak score are shown in Supplementary Figures S2A–D. Logistic regression analysis showed that DNTTIP1 was significantly correlated with T stage (*p* = 0.038), histologic grade (*p* < 0.001), fibrosis Ishak score (*p* = 0.007), vascular invasion (*p* = 0.026), and TP53 status (*p* < 0.001) (Table 2).

**TABLE 2:** DNTTIP1 expression associated with clinicopathological characteristics (logistic regression).

Characteristics	Odds ratio in DNTTIP1 expression	Odds ratio (OR)	*p*-value
T stage (T2 and T3 and T4 vs. T1)	368	1.55 (1.03–2.34)	0.038
N stage (N1 vs. N0)	256	2.95 (0.37–60.13)	0.351
M stage (M1 vs. M0)	270	0.30 (0.01–2.38)	0.300
Pathologic stage (Stage II and Stage III and Stage IV vs. Stage I)	347	1.43 (0.94–2.19)	0.096
Histologic grade (G3 and G4 vs. G1 and G2)	366	2.41 (1.56–3.76)	<0.001
Residual tumor (R1 and R2 vs. R0)	342	1.05 (0.40–2.76)	0.919
Fibrosis Ishak score (1/2 and 3/4 and 5/6 vs. 0)	212	2.30 (1.27–4.24)	0.007
Adjacent hepatic tissue inflammation (mild and severe vs. none)	234	1.53 (0.91–2.58)	0.113
Vascular invasion (yes vs. no)	315	1.70 (1.07–2.73)	0.026
Tumor status (with tumor vs. tumor-free)	352	1.18 (0.78–1.81)	0.433
TP53 status (Mut vs. WT)	358	4.06 (2.48–6.79)	<0.001

**FIGURE 5:**
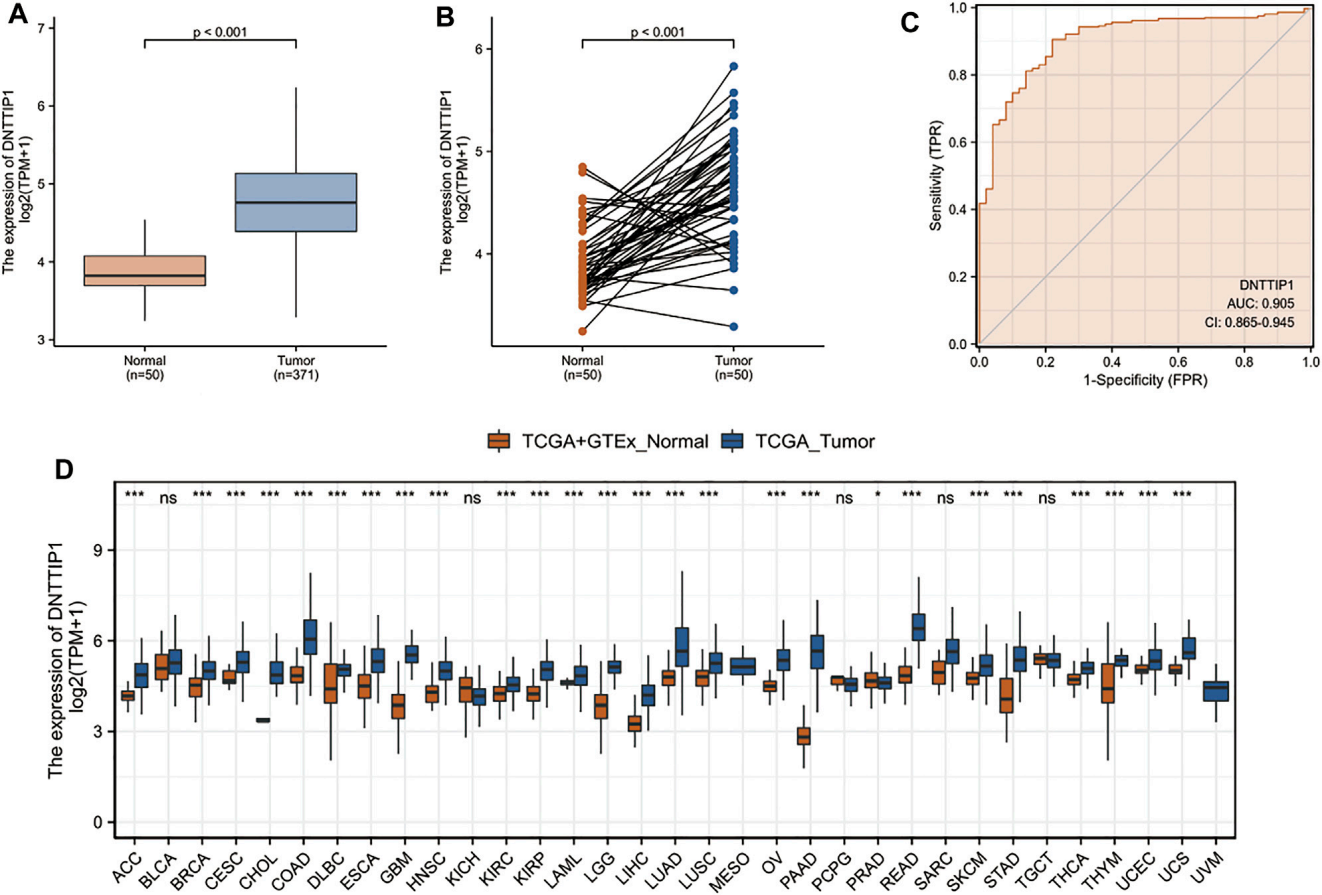
Prognostic value of DNTTIP1 in LIHC and other cancer types. **(A,B)** DNTTIP1 is highly expressed in LIHC samples when compared with normal tissues. **(C)** ROC curve indicates that DNTTIP1 is a potential diagnostic marker. **(D)** DNTTIP1 is significantly overexpressed in other cancer types when compared with normal tissues. Data were processed TPM format RNA-seq data originated from TCGA and GTEx downloaded from UCSC XENA (https://xenabrowser.net/datapages/).

DNTTIP1 is highly expressed in LIHC samples when compared with normal tissues (Figures 5A,B). The area under the curve (AUC) of DNTTIP1 was 0.905, which indicated that DNTTIP1 might be a potential diagnostic molecule (Figure 5C). In many other cancer types such as adrenocortical carcinoma (ACC), breast invasive carcinoma (BRCA), cervical squamous cell carcinoma, and endocervical adenocarcinoma (CESC), DNTTIP1 was also significantly overexpressed when compared with normal tissues (Figure 5D).

In the Cox regression model, variables with *p* < 0.1 in univariate Cox regression were included in multivariate Cox regression. The variables that met this threshold were T stage (*p* < 0.001), M stage (*p* = 0.018), pathologic stage (*p* < 0.001), tumor status (*p* < 0.001), and DNTTIP1 (*p* < 0.001) (Table 3). Furthermore, multivariate Cox regression showed that tumor status (*p* = 0.001) and DNTTIP1 (*p* = 0.027) were independent prognostic factors for overall survival (*p* < 0.05).

**TABLE 3:** Univariate and multivariate analyses of clinicopathological parameters in patients with hepatocellular carcinoma in TCGA–LIHC.

Characteristics	Total (*N*)	HR (95%CI) univariate analysis	*p*-value univariate analysis	HR (95%CI) multivariate analysis	*p*-value multivariate analysis
T stage (T2 and T3 and T4 vs. T1)	367	2.109 (1.469–3.028)	<0.001	0.890 (0.120–6.620)	0.909
N stage (N1 vs. N0)	256	2.004 (0.491–8.181)	0.333		
M stage (M1 vs. M0)	270	4.032 (1.267–12.831)	0.018	1.681 (0.400–7.063)	0.478
Pathologic stage (Stage II and Stage III and Stage IV vs. Stage I)	346	2.074 (1.418–3.032)	<0.001	2.422 (0.314–18.705)	0.396
Histologic grade (G3 and G4 vs. G1 and G2)	365	1.120 (0.781–1.606)	0.539		
Residual tumor (R1 and R2 vs. R0)	341	1.571 (0.795–3.104)	0.194		
Age (>60 vs. ≤60)	370	1.248 (0.880–1.768)	0.214		
Gender (male vs. female)	370	0.816 (0.573–1.163)	0.260		
AFP (ng/ml) (>400 vs. ≤400)	277	1.056 (0.646–1.727)	0.827		
Albumin (g/dl) (≥3.5 vs. <3.5)	296	0.921 (0.565–1.503)	0.743		
Prothrombin time (>4 vs. ≤4)	293	1.330 (0.877–2.015)	0.179		
Fibrosis Ishak score (1/2 and 3/4 & 5/6 vs. 0)	211	0.779 (0.470–1.293)	0.334		
Vascular invasion (yes vs. no)	314	1.348 (0.890–2.042)	0.159		
Tumor status (with tumor vs. tumor-free)	351	2.361 (1.620–3.441)	<0.001	2.299 (1.400–3.774)	0.001
TP53 status (Mut vs. WT)	357	1.434 (0.972–2.115)	0.069	1.393 (0.822–2.359)	0.218
DNTTIP1 (high vs. low)	370	1.941 (1.363–2.764)	<0.001	1.735 (1.064–2.829)	0.027

HR, hazard ratio; CI, confidence interval; AFP, alpha-fetoprotein.

**FIGURE 6:**
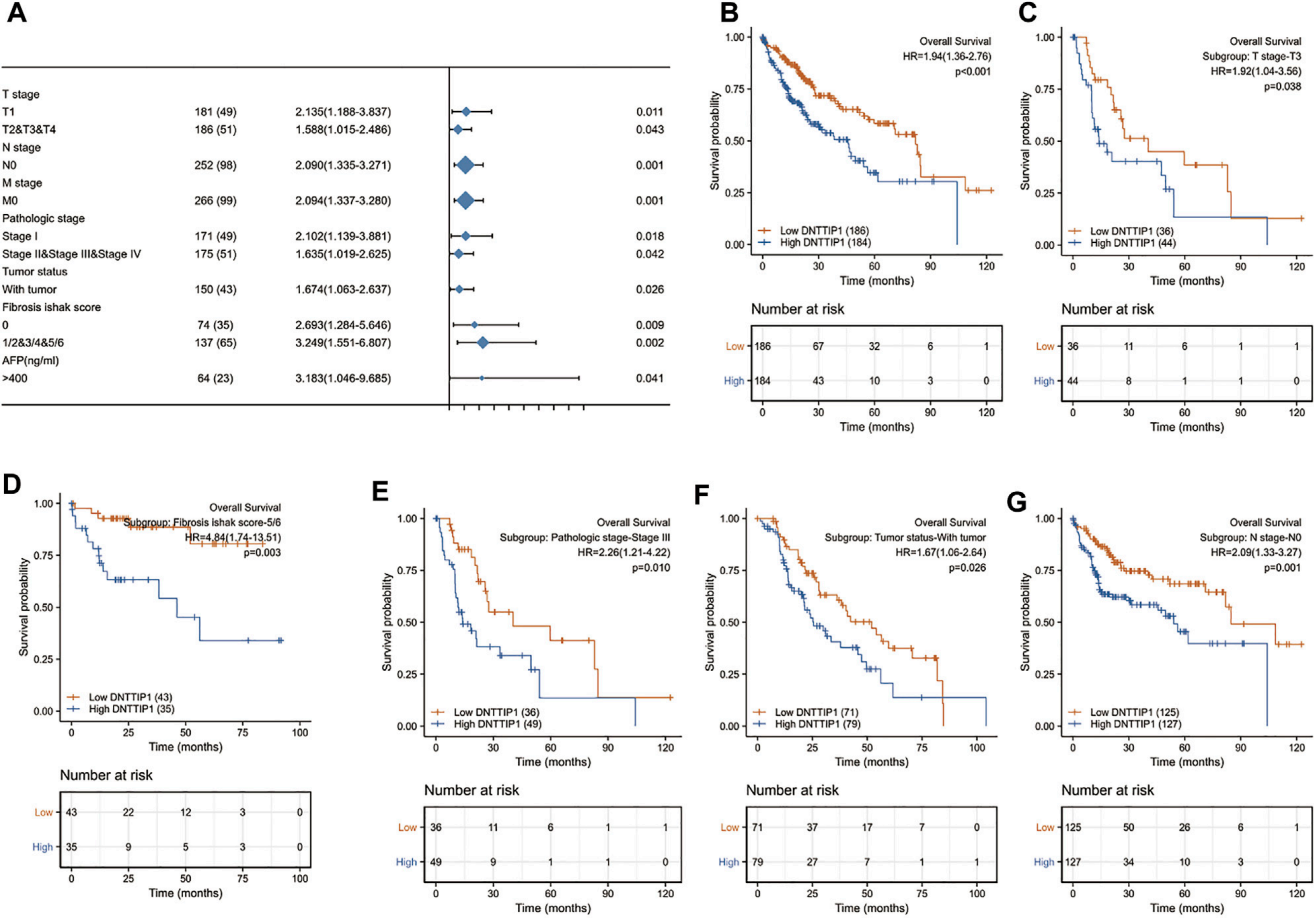
Prognostic value of DNTTIP1 in different analyses. **(A)** Prognostic value of DNTTIP1 in overall survival of HCC. **(B)** Forest plot of the prognostic value of DNTTIP1 in overall survival in different subgroups of HCC in TCGA–LIHC. **(C–G)** High expression of DNTTIP1 was associated with poor overall survival in different subgroups.

The Kaplan–Meier survival curve drawn by the survminer package in R was used to evaluate the prognostic value of DNTTIP1 in overall survival of HCC (Figure 6A). Gene expression values were divided into high- and low-expression groups according to the median value. High expression of DNTTIP1 was associated with poor overall survival (HR = 1.94 (1.36–2.76), *p* < 0.001). The lower part of these figures is shown in the risk table which records the number of people still under follow-up at each time point. The prognosis data are derived from an article published in Cell (Liu et al., 2018).

Based on multivariate Cox regression, a nomogram that integrated DNTTIP1 and independent clinical risk factors (tumor status and DNTTIP1) was drawn by using the rms package in R as a tool for clinicians to predict the prognosis of HCC patients (Supplementary Figure S3A). The C-index in the nomogram defines the accuracy of the model. C-index values are generally between 0.5–1. The main contents of the nomogram are as follows: the variables of the prediction model (scales are marked on the line segment corresponding to each variable, which represent the range of the variable, while the length of the line segment reflects the contribution of the factor to the outcome events); score (the individual score of each variable under different values and the total score of the sum of the individual scores corresponding to the value of all variables); prediction probability (survival probability at different time nodes). Predictions made by the calibration curve conformed well to the ideal line (the 45-degree line), which indicated that the prediction was consistent with the observation (Supplementary Figure S3B).

The prognostic values of DNTTIP1 in relation to the overall survival under different subgroups of LIHC in TCGA are shown in Table 2. The expression of DNTTIP1 had significant effect in T1 (HR = 2.135 (1.188–3.837), *p* = 0.011), T2, and T3 and T4 (HR = 1.588 (1.015–2.486, *p* = 0.043) subgroups of T stage, N0 (HR = 2.090 (1.335–3.271, *p* = 0.001) subgroups of N stage, and M0 (HR = 2.094 (1.337–3.280), *p* = 0.001) of M stage (Figure 6B). High expression of DNTTIP1 was associated with poor overall survival in the T3 subgroup of T stage (HR = 1.92 (1.04–3.56), *p* = 0.038), 5/6 subgroup of fibrosis Ishak score (HR = 4.84 (1.74–13.51), *p* = 0.003), Stage III subgroup of pathologic stage (HR = 2.26 (1.21–4.22), *p* = 0.010), with tumor subgroup of tumor status (HR = 1.92 (1.04–3.56), *p* = 0.038), and N0 subgroup of N stage (HR = 2.09 (1.33–3.27), *p* = 0.001) (Figures 6C–G).

## Discussion

Clinicians have been perplexed for a long time by the early detection of HCC that adopts AFP as an indicator for HCC screening at the early stage. More than 30% of HCC patients are AFP negative (Luo et al., 2018). Therefore, new markers for HCC are needed to improve early diagnosis.

Previous *in vitro* and *in vivo* studies have shown that DNTTIP1 interacts with histone deacetylase 1 (HDAC1) to control the status of p53 acetylation (Sawai et al., 2018), thus regulating the expression of several p53 target genes that participate in cell cycle arrest (Narayanan et al., 2003; Zhang et al., 2004) and promote tumor cell migration and invasion in oral squamous cell carcinomas (OSCCs). Former bioinformatics analysis indicated that DNTTIP1 could predict the survival of patients with acute myelocytic leukemia (Zhuang et al., 2020). Yet, the role of DNTTIP1 in HCC progression needs to be further investigated.

In this study, bioinformatics analysis of sequencing data from TCGA was performed to gain a deeper understanding of the potential function of DNTTIP1 in HCC and guide future research in HCC. RNA-seq normalization was realized to remove the technical deviation of sequencing data: sequencing depth and gene length. We transferred count data to TPM. This RNA-seq normalization step not only eliminated the influence of single sequencing depth and gene length but also TPM quantified gene expression rate so that the data can be compared among cells with different basal expressions.

Elevated DNTTIP1 expression in HCC was associated with advanced clinicopathological features (AFP, fibrosis Ishak score, histologic grade, pathological stage, TP53 status, and vascular invasion), poor prognosis, and survival time. Furthermore, in univariate and multivariate Cox regression analyses, we found that after removing confounding factors, DNTTIP1 was still an independent prognostic factor, which showed a higher prognostic value than many other clinical variables, including AFP. Our results suggested that DNTTIP1 is a potential prognostic and diagnostic marker deserving further clinical validation. The function of DNTTIP1 in HCC was further investigated in GSEA using TCGA data.

The PPI network indicates that DNTTIP1 can interact with several histone deacetylase (HDAC) family members other than HDAC1. HDACs are key enzymes that maintain the acetylation balance of nucleosomes in basic units of chromosomes. Their catalytic histone deacetylation is closely related to the inhibition of gene transcription (Sanaei and Kavoosi, 2019). GSEA showed that cell cycle mitotic, G1/S, and G2/M phases and Fc fragment of IgE receptor I (FCERI)–mediated NF-κB and MAPK pathway and Fc fragment of IgG receptor (FCGR) activation in HCC were enriched in the DNTTIP1 high-expression phenotype. These findings indicated that DNTTIP1 might participate in the regulation of cell cycle and immune response in the tumorigenesis of HCCs.

SSGSEA and Spearman’s correlation were adopted to uncover connections between DNTTIP1 expression and immune infiltration levels in HCC. Our results demonstrated that DNTTIP1 expression was significantly positively correlated with Th2 cells. Furthermore, there was a strong-to-moderate correlation between Tfh, NK CD56 bright cells, aDCs, T helper cells, Th1 cells, macrophages, and DNTTIP1 expression. Our results suggest a possible mechanism where DNTTIP1 regulates the balance of Th1/Th2 in HCC. The Th2 cells produce IL-4 and IL-10 and inhibit the host immune system, hence having a role in promoting tumor growth (Ko et al., 2001; Zhao et al., 2015). This indicates that overexpression of DNTTIP1 promotes Th2 cell immune response and infiltration in tumor progression. Th1/Th2 balance can be regulated to inhibit tumor progression. A global Th1/Th2-like cytokine shift (a decrease in Th1 and an increase in Th2 cytokines) can be induced to promote HCC metastasis (Budhu et al., 2006).

On the other hand, there was an inverse correlation between Th17 immune cells, neutrophils, DCs, and DNTTIP1. Th17 immune cells were associated with both good and bad prognoses (Guéry and Hugues, 2015). It is reported that Th17 cells were correlated with a bad prognosis of HCC (Zhang et al., 2009). However, Th17 cells can also drive antitumor immune responses by recruiting immune cells into tumors, activating effector CD8^+^ T cells, or even directly by converting toward Th1 phenotype and producing IFN-γ (Lee et al., 2009; Nistala et al., 2010). The downregulation of Th17 caused by DNTTIP1 overexpression may affect h1/h2 balance, leading to a bad prognosis. Neutrophils have an active role in regulating the immune system; they can promote or inhibit the establishment of a permissive tumor microenvironment (Shaul and Fridlender, 2017). Many studies have shown that tumor-associated neutrophils not only promote tumor growth (Wang et al., 2020) but also antitumor effects on tumors and can regulate their different phenotypes through tumor signal transduction. Our result indicates that the antitumor function of neutrophils may be hindered when DNTTIP1 is overexpressed. Most DC subsets have been found in tumors where they play a major role in cancer immune surveillance by coordinating adaptive immunity against tumor antigens. DCs are critical for autoimmunity and tissue inflammation and have prominent roles in cellular and humoral immune response and protection from infectious diseases or tumors (Vatner and Janssen, 2019). Due to the role of DCs in initiating antitumor immunity, there is a negative selective pressure hampering the accumulation of DCs by tumor-secreted mediators that inhibit dendropoiesis, promote DC apoptosis (Pirtskhalaishvili et al., 2000), and accelerate DC turnover. The suppression of DCs is normally found in tumors and may facilitate HCC progression. All findings according to ssGSEA support that DNTTIP1 has a role in regulating and recruiting immune infiltrating cells in HCC. However, more trials are needed to accurately understand the relationship between DNTTIP1 and Th1/Th2 balance *in vivo*.

In addition, the Kaplan–Meier survival curves with high HR for poor OS and PFS when DNTTIP1 was highly expressed in HCC showed the correlation between high-level expression of DNTTIP1 and poor prognosis of HCC, thus suggesting that DNTTIP1 was a prognostic biomarker in HCC.

The Cox HR model suggested that DNTTIP1 was strongly associated with OS in patients. Moreover, we developed a DNTTIP1-related nomogram to predict the 1-, 3-, and 5-year survival probabilities in HCC patients. The results were confirmed by calibration plots and log-rank tests.

Moreover, Kaplan–Meier survival analysis was performed in HCC patients according to their DNTTIP1 expression levels, stratified by clinicopathological characteristics. A high level of DNTTIP1 expression was associated with poor prognosis of HCC patients with a fibrosis Ishak score of 5/6, thus indicating that the high association between DNTTIP1 expression level and survival may be influenced by the degree of hepatic fibrosis.

Although our investigation of the relationship between DNTTIP1 and HCC furthered our understanding of the vital role of DNTTIP1 in HCC, some limitations remained. First, cell experiments and clinical samples should be used to verify the correlation between DNTTIP1 mRNA and protein expression. In the present study, we only used mRNA levels to predict protein expression (Fortelny et al., 2017). Second, clinical factors such as the details of patient treatment should be sufficiently considered to clarify the specific role of DNTTIP1 in the development of HCC. Third, while multicenter research based on public databases intends to overcome the shortage of single-center studies, retrospective studies have two major shortages. One is missing variables. In our study, to clarify the specific role of DNTTIP1 in the development of HCC comprehensively, more clinical factors should be taken into consideration such as the detail of treatments for every single patient involved. However, the information of treatments was often inconsistent or even lacking in public databases; the other is sample size imbalance. We have a smaller number of healthy samples in our control group than that of HCC patients in our study; the sample size imbalance may lead to statistical bias. Therefore, future prospective studies are needed to reduce analysis bias. Finally, we cannot illustrate the expression of DNTTIP1 from the protein level and also cannot evaluate the direct mechanisms of DNTTIP1 involved in HCC progression. Consequently, further studies are needed to clarify the direct mechanisms of DNTTIP1 in HCC.

In summary, DNTTIP1 has an important role in the regulation of cell cycle and immune response in the tumorigenesis of HCC. Further studies are needed to clarify the biological mechanisms of DNTTIP1 in HCC. In addition, additional experiments are needed to evaluate the relationship between DNTTIP1 expression and clinical features, HCC stage, and prognosis using additional clinical data, which might facilitate the identification of new markers for evaluating tumor stage, aiding drug development, and improving treatment efficiency.

## Data Availability

The datasets presented in this study can be found in online repositories. The names of the repository/repositories and accession number(s) can be found in the article/Supplementary Material.

## Supplementary Material

Supplementary Figure 1 Volcano plot of differentially expressed RNAs. **(A)** Volcano plot of differentially expressed mRNAs. **(B)** Volcano plot of differentially expressed lncRNAs.

Supplementary Figure 2 Association between DNTTIP1 expression and different clinicopathological characteristics after *post hoc* Dunn’s test with Bonferroni correction for pairwise comparisons. **(A)** Association between DNTTIP1 expression and the T stage of HCC, **(B)** pathologic stage, **(C)** histologic grade, and **(D)** Fibrosis Ishak score.

Supplementary Figure 3 Nomogram and calibration curve. **(A)** Nomogram that integrated DNTTIP1 and independent clinical risk factors between tumor status and DNTTIP1. **(B)** Predictions made by the calibration curve were found to conform well to the ideal line.
