# Supplementary material for: DNTTIP1 is a Prognostic Biomarker Correlated With Immune Infiltrates in Hepatocellular Carcinoma: A Study Based on The Cancer Genome Atlas Data
Source: Front Genet. 2022 Feb 21;12:767834. doi: 10.3389/fgene.2021.767834 (PMC8899818; doi:10.3389/fgene.2021.767834)
Supplement: Supplementary file 1 [file Table1.docx]

**S1 Table** KEGG Pathways enriched in high‐risk and low‐risk groups by using GSEA

| **Gene set name** | **NES** | **NOM p value** | **FDR q value** |
| --- | --- | --- | --- |
| REACTOME_FCERI_MEDIATED_NF_KB_ACTIVATION | 3.162 | 0.001 | 0.012 |
| REACTOME_FCGR_ACTIVATION | 3.102 | 0.001 | 0.012 |
| REACTOME_FCERI_MEDIATED_CAPLUS2_MOBILIZATION | 3.059 | 0.001 | 0.012 |
| REACTOME_CELL_CYCLE_MITOTIC | 2.527 | 0.001 | 0.012 |
| REACTOME_CELL_CYCLE_CHECKPOINTS | 2.495 | 0.001 | 0.012 |
| REACTOME_MITOTIC_G1_G1_S_PHASES | 2.381 | 0.001 | 0.012 |
| REACTOME_M_PHASE | 2.311 | 0.001 | 0.012 |
| REACTOME_MITOTIC_G2_G2_M_PHASES | 2.308 | 0.001 | 0.012 |

Abbreviations: GSEA, gene set enrichment analysis; NES, normalized enrichment score; NOM, nominal; FDR, false discovery rate. Gene sets with NOM p-val < 0.05 and FDR q-val < 0.25 are considered as significant
