# Supplementary figures and images for: DNTTIP1 is a Prognostic Biomarker Correlated With Immune Infiltrates in Hepatocellular Carcinoma: A Study Based on The Cancer Genome Atlas Data
Source: Front Genet. 2022 Feb 21;12:767834. doi: 10.3389/fgene.2021.767834 (PMC8899818; doi:10.3389/fgene.2021.767834)

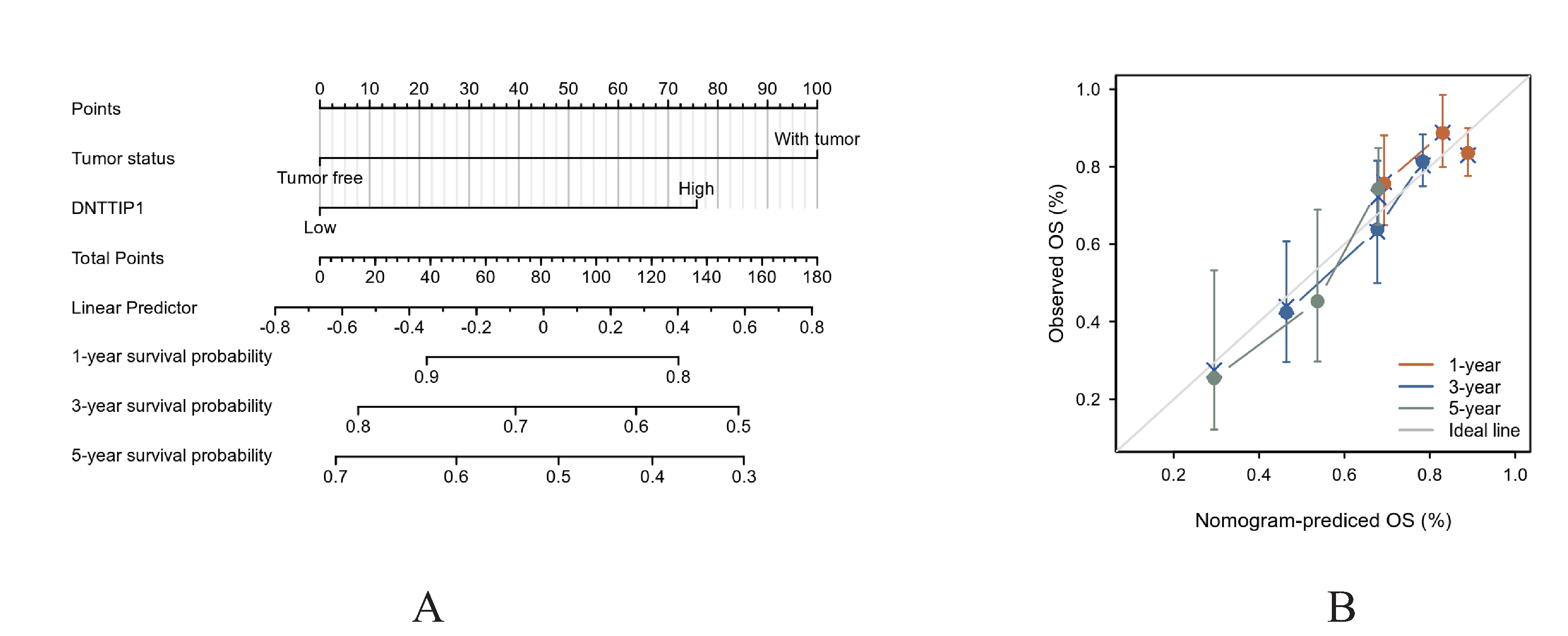

Supplement: Supplementary file 2 [file Image3.TIF]

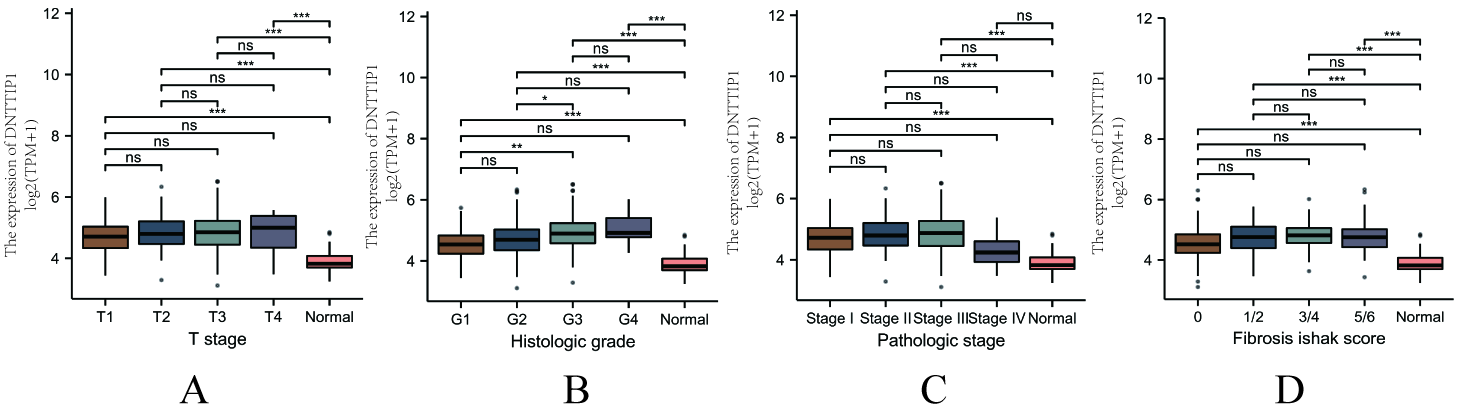

Supplement: Supplementary file 3 [file Image2.TIF]

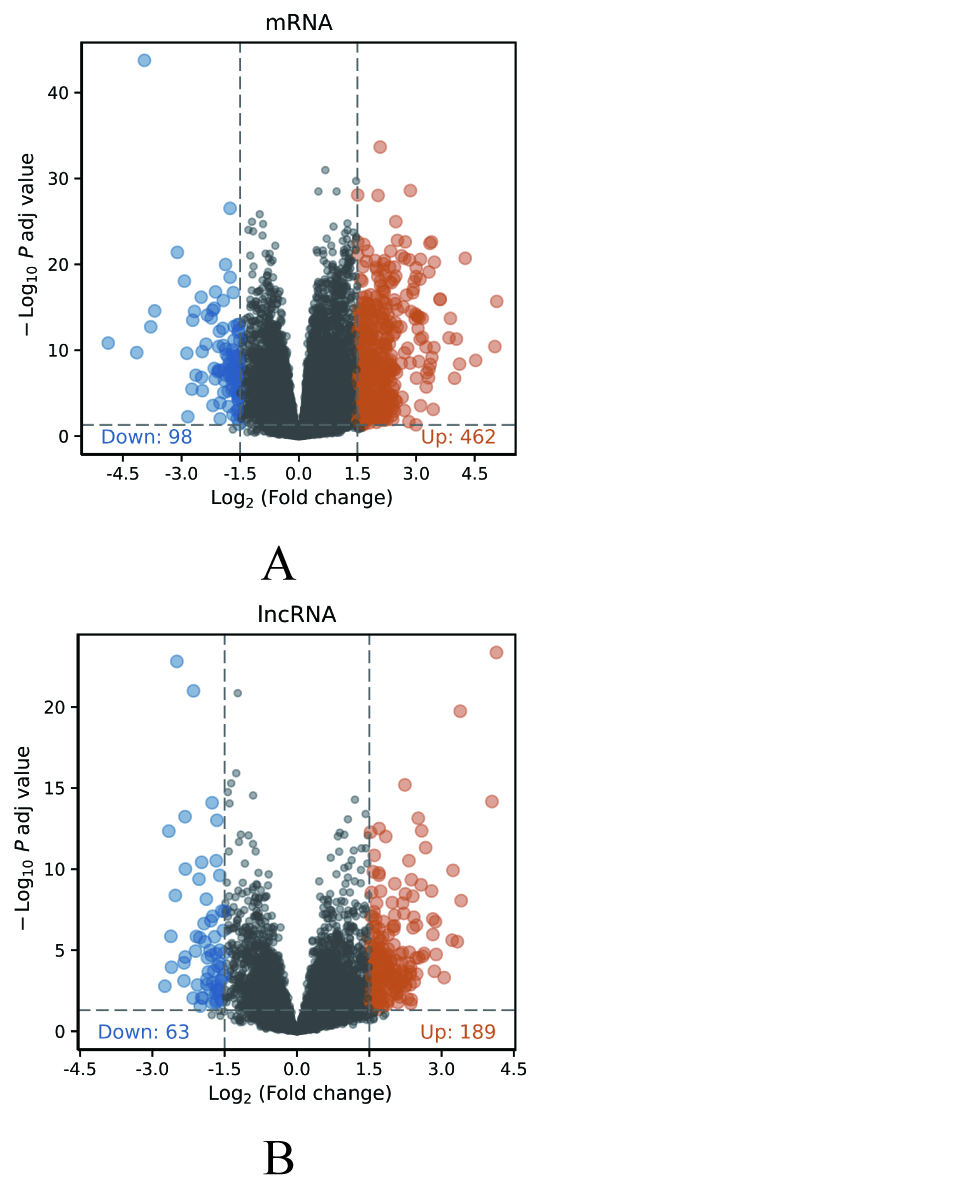

Supplement: Supplementary file 4 [file Image1.TIF]
